# Onasemnogene abeparvovec in spinal muscular atrophy: an Australian experience of safety and efficacy

**DOI:** 10.1002/acn3.51519

**Published:** 2022-02-16

**Authors:** Arlene M. D'Silva, Sandra Holland, Didu Kariyawasam, Karen Herbert, Peter Barclay, Anita Cairns, Suzanna C. MacLennan, Monique M. Ryan, Hugo Sampaio, Nicholas Smith, Ian R. Woodcock, Eppie M. Yiu, Ian E. Alexander, Michelle A. Farrar

**Affiliations:** ^1^ Department of Neurology Sydney Children's Hospital Network Sydney New South Wales Australia; ^2^ School of Clinical Medicine, UNSW Medicine & Health, Randwick Clinical Campus, Discipline of Paediatrics University of New South Wales Sydney New South Wales Australia; ^3^ Physiotherapy Sydney Children's Hospital Sydney New South Wales Australia; ^4^ Department of Pharmacy Prince of Wales Hospital, Sydney Children's Hospital Randwick and The Royal Hospital for Women Randwick New South Wales Australia; ^5^ Neurosciences Department Queensland's Children Hospital South Brisbane Queensland Australia; ^6^ Neurology Department Women's & Children's Hospital North Adelaide South Australia Australia; ^7^ School of Paediatrics and Reproductive Health University of Adelaide Adelaide South Australia Australia; ^8^ Department of Neurology Royal Children's Hospital Melbourne Australia; ^9^ Neuroscience Research Group Murdoch Children's Research Institute Melbourne Australia; ^10^ Department of Paediatrics The University of Melbourne Melbourne Australia; ^11^ Gene Therapy Research Unit Sydney Children's Hospitals Network and Children's Medical Research Institute, The University of Sydney Westmead Australia; ^12^ Discipline of Child and Adolescent Health, Sydney Medical School, Faculty of Medicine and Health The University of Sydney Sydney New South Wales Australia

## Abstract

**Objective:**

To provide a greater understanding of the tolerability, safety and clinical outcomes of onasemnogene abeparvovec in real‐world practice, in a broad population of infants with spinal muscular atrophy (SMA).

**Methods:**

A prospective cohort study of children with SMA treated with onasemnogene abeparvovec at Sydney Children's Hospital Network, Australia was conducted from August 2019 to November 2021. Safety outcomes included clinical and laboratory evaluations. Efficacy assessments included World Health Organisation (WHO) motor milestones, oral and swallowing abilities, and requirements for respiratory support. The implementation of a model of care for onasemnogene abeparvovec administration in health practice is described.

**Results:**

21 children were treated (age range, 0.65–24 months; body weight range, 2.5–12.5 kg) and 19/21 (90.4%) had previous nusinersen. Transient treatment‐related side effects occurred in all children; vomiting (100%), transaminitis (57%) and thrombocytopaenia (33%). Incidence of moderate/severe transaminitis was significantly greater in infants weighing ≥8 kg compared with <8 kg (*p* < 0.05). Duration of prednisolone following treatment was prolonged (mean 87.5 days, range 57–274 days). 16/21 (76%) children gained at least one WHO motor milestone. Stabilisation or improvement in bulbar or respiratory function was observed in 20/21 (95.2%) patients. Implementation challenges were mitigated by developing standard operating procedures and facilitating exchange of knowledge.

**Interpretation:**

This study provides real‐world evidence to inform treatment decisions and guide therapeutic expectations for onasemnogene abeparvovec and combination therapy for SMA in health practice, especially for children weighing ≥8 kg receiving higher vector loads. Proactive clinical and laboratory surveillance is essential to facilitate individualised management of risks.

## Introduction

The therapeutic landscape for neurodegenerative diseases has been revolutionised by the recent introduction of genetic molecular technologies. Spinal muscular atrophy (SMA), previously the leading genetic cause of infant mortality, is the archetypal disease where the application of viral vector‐mediated gene therapy has changed the natural course of the condition. Caused by biallelic mutations of the *survival motor neuron 1* (*SMN1*) gene, leading to deficiency in functional survival motor neuron (SMN) protein, SMA is characterised by progressive limb muscle weakness and atrophy leading to a decline in motor function and accumulation of comorbidities including bulbar difficulties and respiratory insufficiency.^1^

The spectrum of severity at diagnosis ranges from presymptomatic newborns to infantile onset SMA, characterized by severe hypotonia and weakness before 6 months of age with an inability to achieve unsupported sitting. Childhood onset forms meanwhile are characterised by a gradual loss in motor skills over time. Phenotypic severity and age of onset vary inversely with *survival motor neuron 2* (*SMN2)* copy number.^2^

Advanced molecular therapeutic strategies target repletion of SMN protein. Under this umbrella of SMN‐dependent therapies, nusinersen (Spinraza^®^), an intrathecally administered anti‐sense oligonucleotide (ASO) alters the splicing of SMN2 pre‐mRNA and increases SMN levels.^3^ Nusinersen was approved and reimbursed in Australia from 2018 for symptomatic children aged <18 years. In contrast, onasemnogene abeparvovec (Zolgensma^®^) delivers a functional human *SMN* cDNA transgene via a non‐replicating recombinant adeno‐associated virus pseudo‐serotype with the type 9 capsid presenting the ability to cross the blood brain barrier following systemic delivery.^4^ This therapeutic agent gained therapeutic goods administration (TGA) approval in Australia for the treatment of paediatric patients <9 months of age with symptomatic or presymptomatic SMA from February 2021. However, it is not yet funded under the Pharmaceutical Benefits Scheme (PBS). Risdiplam is an orally administered small molecule modifying *SMN2* pre–messenger RNA splicing. By August 2021, it had received approval and reimbursement status for the treatment of symptomatic individuals aged 2 months to 18 years in Australia.

Thus far, published clinical trials and real‐world data for the safe and efficacious administration of onasemnogene abeparvovec has focussed on monotherapy in homogeneous and narrow populations, limiting the generalisability of treatment outcomes.^4^, ^5^, ^6^, ^7^, ^8^ Most trials have included treatment‐naïve children aged ≤6 months and weighing below 8.4 kg.^4^, ^8^ Those trials have shown improvements in survival and respiratory and bulbar function and attainment of motor milestones, especially in children treated presymptomatically. Gene therapy vector dose is weight‐dependent; the safety and efficacy of onasemnogene abeparvovec in a more heterogeneous population of older, heavier and/or symptomatic children receiving combination or sequential therapies is less well‐defined and requires interrogation. In addition, as the therapeutic paradigm shifts and more treatment options emerge, the focus for clinicians and families now goes beyond just survival and motor function scores. Decision‐making for therapeutic approach is thus influenced by additional benefits that are patient‐ and family‐driven. These include stability or improvements in feeding, swallowing, fatigue, respiratory and motor function, together with reduced long‐term burden of treatment.^9^

This study aims to provide a greater understanding of the tolerability, safety and clinical outcomes of onasemnogene abeparvovec in real‐world practice, including a broad population of infants with SMA. As a novel therapeutic, the logistics of establishing service provision for safe, equitable and efficient administration of onasemnogene abeparvovec is complex. Importantly, this study also describes the clinical translation of a model of care for SMA patients, including challenges associated with establishing appropriate infrastructure, delivery and medical management during the COVID‐19 pandemic, across multiple jurisdictions.

## Subjects/Materials and Methods

### Study participants and setting

This was a cohort study of children who received onasemnogene abeparvovec from August 2019–November 2021 at Sydney Children's Hospital Network (SCHN), Australia. As one of the clinical trial sites for onasemnogene abeparvovec treatment, SCHN developed health system readiness and was approved by Novartis Gene Therapies as a treatment centre for the onasemnogene abeparvovec Global Managed Access Program (GMAP), receiving referrals by neurologists from national tertiary neuromuscular centres. The purpose of the GMAP was to make the investigational drug available for those with serious disease whilst national regulatory and reimbursement approvals were undertaken. Patient access to onasemnogene abeparvovec was obtained via the TGA and costs associated with the drug were funded through industry, federal government or by individual means.

Inclusion criteria were biallelic mutations in *SMN1*, age < 2 years, normal range baseline blood values including, but not limited to platelet count, total bilirubin, liver transaminases, prothrombin time, white cell count, creatine kinase and anti‐AAV9 antibody titres ≤1:50. *SMN2* copy number was ascertained but not a specific inclusion or exclusion criteria. The patient pathway to access onasemnogene abeparvovec was established, and eligibility determined by multiple stakeholders.

The study was approved by the Sydney Children's Hospitals Network Human Research Ethics Committee (2020/ETH03152). Written and informed consent for onasemnogene abeparvovec administration alongside use of patient data was obtained from parents prior to the study.

### Administration of gene therapy and follow up

Infants received a single dose of intravenous (IV) onasemnogene abeparvovec (1.1 × 10^14^ vg/kg) over 60 min, on day one of the treatment period. Initially, children were not dosed within 1 week of immunisation.^10^ The timeline between vaccination/recent illness and dosing was subsequently increased to 4 weeks following a serious adverse event in one of our first patients.

Post infusion, patients were monitored for a period of 2–4 h, discharged if stable and reviewed within 24 h as an outpatient. Dosage and administration was in line with the FDA prescribing information.^11^ Specifically, all infants commenced oral prednisolone (1 mg/kg/day) the day prior to infusion. Weekly clinical assessments and laboratory testing of liver function [alanine aminostransferase (ALT), aspartate aminostransferase (AST), bilirubin and gamma‐glutamyl transferase (GGT)], full blood count and renal function were undertaken for the first month. Prednisolone was continued for a minimum of 30 days then weaned, providing ALT and AST concentrations were less than two times upper limit of normal (ULN) values. Routine immunisations were withheld for 30 days post‐cessation of prednisolone. Because of the theoretical risk of insertional oncogenesis in the liver, abdominal ultrasounds and serum alpha‐fetoprotein concentrations were conducted annually (and are ongoing). Baseline electrocardiogram (ECG) and blood troponin I concentrations were recorded prior to dosing and continued every 3 months for a period of 12 months post‐administration.

### Clinical data and outcome measures

Clinical characteristics, laboratory, SMA management and caregiver‐reported rationale for therapeutic choices were collated from medical records. Follow up was from the time of onasemnogene abeparvovec administration (baseline) until November 2021.

Respiratory, feeding and swallowing function included ventilatory requirements and bulbar function was assessed clinically with history of fatigue, choking or coughing episodes with feeding, alongside assessment of weight. Swallow studies were undertaken when clinical indicators were present and guided implementation of supplemental nutrition (nasogastric, nasojejunal or gastrostomy tube). The Oral and Swallowing Abilities Tool (OrSAT) was applied through retrospective analysis of patient records to provide an objective measure of bulbar function at baseline and follow up.^12^ This is a checklist that uses questions asked routinely in a clinical setting, relevant to SMA and developmentally appropriate infants, including an infant's ability to take different food consistencies, need for intervention, behaviour during meals, speech and degree of impairment.

Clinicians assessed attainment of gross motor development milestones (sitting without support, standing with assistance, hands‐and‐knees crawling, walking with assistance, standing alone and walking alone) by World Health Organization (WHO) criteria^13^ and specific items on the Bayley Scale of Infant and Toddler Development III (BSID‐III). Specifically, the ability to sit, walk with support and walk independently (Items 26, 37 and 34) were recorded through the study intervals. Validated assessments for use in children with SMA were conducted by a dedicated trained neuromuscular physiotherapist and included the Children's Hospital of Philadelphia Infant Test of Neuromuscular Disorders (CHOP INTEND) and Hammersmith Functional Motor Scale/extended version (HFMSE) for those who had demonstrated at minimum, the ability to sit. In both assessments, higher scores indicate better motor function.

### Clinical translation of a model of care for safe and efficient administration of onasemnogene abeparvovec to SMA patients

Challenges and implementation actions to develop a model of care for onasemnogene abeparvovec administration within an established neuromuscular service framework was analysed. Facilitators and barriers associated with logistics, delivery and medical management were identified within the context of the COVID‐19 pandemic. The infrastructure required to manage multiple stakeholders within the patient pathway and streamline unified and equitable access to onasemnogene abeparvovec across a heterogenous demographic was evaluated and documented.

### Data analysis

Patient data from electronic medical records were imported into the IBM Statistical Package for the Social Sciences (SPSS®) Statistics 25 software for data analysis. Descriptive statistics, including frequencies and percentages, medians with ranges were reported. Elevated transaminases (AST/ALT) included concentrations above the laboratory upper limit of normal of 36 U/L and categorised as mild (3–5 × ULN), moderate (5–20 × ULN) and severe (≥20 × ULN). 16 ng/L.Elevated bilbirubin and GGT concentrations included those above a laboratory ULN of 20 and 50 μmol/L respectively. Subgroup analysis based on age (≥12–24 months and <12 months) and weight (≥8 kg and < 8 kg) was undertaken. Elevation of AST and ALT were compared by age and weight categories using the chi‐squared test. Plots were generated using GraphPad Prism 8 to assess the magnitude of elevations in laboratory measures. The challenges and implementation actions of the program were analysed using an iterative methodology using real‐time feedback from stakeholders.

## Results

### Subject demographics and baseline characteristics

The cohort included 21 children (7 males, 14 females). Infants lived across five states and territories of Australia, a geographical area comparable to one‐third of the United States. The average distance travelled for onasemnogene abeparvovec treatment was 533 km (range 21–1379 km) (Table 1).

**Table 1:** Demographics and clinical characteristics of children treated with onasemnogene abeparvovec.

Cohort characteristics	*n* = 21
Sex	
Male	7 (33)
Female	14 (67)
Modality of diagnosis	
Clinical symptoms	10 (47.6)
Newborn screening	11 (52.4)
*SMN2* genotype	
Two copies	16 (76)
Three copies	5 (14)
Median age (range) at diagnosis, days	27 (9–329)
Median age (range) at dosing, months	11 (0.65–24)
Age <6 months	3
Age ≥6 to <12 months	8
Age ≥12 to <24 months	10
Weight ≥8 kg	15
Weight <8 kg	6
Median weight (range) at dosing, kg	8.8 (2.5–12.5)
Wt/kgs <6 months	6.2 (2.5–7.7)
Wt/kgs ≥6 to <12 months	8.0 (6.7–9.2)
Wt/kgs ≥12 to <24 months	9.8 (8.7–12.5)
SMA phenotype at dosing	
Non‐sitter	14 (66.7%)
Sitter	3 (14.3%)
Walker	2 (9/5%)
Caregiver reasons for onaemnogene abeparvovec	
Hopes of additional benefit	21
Previous treatment response: lack of efficacy	0
Previous treatment with nusinersen, n (%)	19/21 (90.4%)
Median number (range) of nusinersen injections prior to onasemnogene abeparvovec	5 (3–7)
Distance travelled from home to SCH Median distance, kms (range)	533 (21–1379)

Values expressed as number (%) unless otherwise stated.

Of the cohort, 3/21 (14.3%) were <6 months with a median weight of 6.2 kg (2.5–7.7), 8/21 (38%) were ≥6 to <12 months of age with a median average weight 8 kg (6.7–9.2 kg), and 10/21(47.6%) were ≥12–24 months of age with a median weight of 9.8 kg (8.7–12.5 kg) at the time of onasemnogene abeparvovec infusion.

In total, 19/21 (90.5%) received onasemnogene abeparvovec following previous nusinersen treatment and two infants (9.5%) had no prior disease‐modifying treatment. No child received risdiplam prior to onasemnogene abeparvovec. Combination therapy with ongoing nusinersen was continued in 5/21 (23.8%) following onasemnogene abeparvovec (Table 1). The median age at dosing for these patients was 11 (range 4–16) months and the median time from onasemnogene abeparvovec to first continued nusinersen for these patients was 3 (range 1–4) months. There was a range of different phenotypes at baseline, from infants with severe SMA (non‐sitters) to developmentally appropriate presymptomatic infants.

Respiratory comorbidities requiring non‐invasive ventilation (NIV) during sleep were present in 6/21 (28.6%) and one infant had a history of hospitalisation and intensive care admission for management of a lower respiratory tract infection, at baseline. Age appropriate oral and swallowing abilities with no impairment of feeding were evident in 14/21 (67%) infants at baseline, while moderate impairment requiring supplemental nutrition (nasogastric, nasojejunal or gastrostomy tube) was present in 1/21 (4.7%) and severe impairment with all nutrition and hydration through non‐oral means was present in 6/21 (28.5%).

### Caregiver rationale for accessing onasemnogene abeparvovec

Parental preference for a single IV injection and hopes for additional clinical benefits such as gains in function, reduction in fatigue and improved endurance were the major reasons for accessing onasemnogene abeparvovec. Those previously treated with nusinersen had shown gains in motor function, but similar gains in respiratory and swallow function were not evident in infants with severe phenotypes. All parents, particularly parents of symptomatic children (14/21; 67%), cited hopes for improvements in feeding abilities and reduction in hospitalisations due to respiratory illnesses and ventilatory support time; both major causes of morbidity and mortality in SMA (Table 1). Five infants with lower baseline motor, bulbar and respiratory function, received ongoing combination therapy with nusinersen and onasemnogene abeparvovec, seeking to maximise functional improvements.

### Safety outcomes for onasemnogene abeparvovec

**Figure 1:**
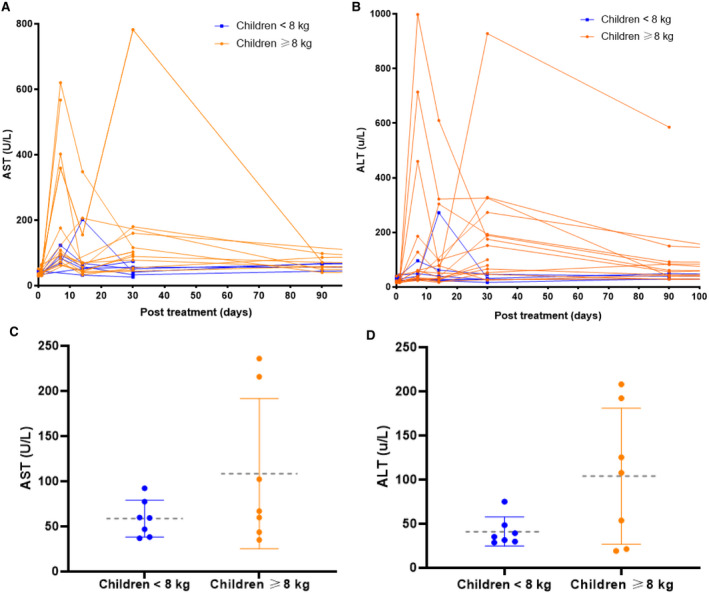
(A) Asparatate aminotransferase (AST) and (B) Alanine Aminotransferase (ALT) (number of times above the upper limit of normal) following onasemnogene dosing. Mean (circles) with standard deviation (dashed grey line) in (C) AST and (D) ALT following onasemnogene dosing. Children weighing ≥8 kg are represented by orange lines and children weighing <8 kg are represented by blue lines. [Colour figure can be viewed at wileyonlinelibrary.com]

The most common treatment‐related adverse events were vomiting and elevated aminotransferase concentrations. All infants experienced vomiting, which occurred at a median of 4 days (range 1–8 days) post‐onasemnogene abeparvovec administration and was managed with antiemetic medication and careful maintenance of hydration by oral or enteral feeding. Three infants required hospitalisation for fluid replacement, either via nasogastric or intravenous means and systemic corticosteroid administration. Transaminitis occurred in 12/21 (57%) of the cohort. Elevation of transaminases had two peaks, the first noted days 7 to 14 and/or the second at week four (Fig. 1). Concurrent with the first peak, the incidence of mild, moderate, and severe transaminitis was 3/21 (14%), 7/21 (33%) and 1/21 (4.7%) respectively. At week 4, the incidence of mild, moderate and severe transaminitis was 3/21 (14.2%), 4/21 (19%) and 1/21 (4.8%) respectively. The incidence of moderate and severe transaminitis was greater in infants weighing ≥8 kg (10/15, 66%), compared with infants weighing <8 kg (2/6, 33%), *p* < 0.05. No child had an elevation of bilirubin, and 3/21 (14.3%) children had an elevation of GGT following onasemnogene abeparvovec.

Individualised dosing of prednisolone (2–4 mg/kg/day) occurred in those with moderate and severe transaminitis and weaning, and cessation of prednisolone (when <2xULN) occurred on average 87.5 days (range 57–274 days) post‐onasemnogene abeparvovec. Administration of intravenous pulse (30 mg/kg) methylprednisolone was administered for 3 days in one infant for elevated serum transaminases and normal synthetic function, with no immediate improvement. Post cessation of prednisolone, 17/21 (81%) infants had transaminase concentrations that remained <2xULN, whilst 3/21 (14%) infants experienced a mild transaminitis which was conservatively managed and spontaneously reduced over 3 months. One infant continued on prednisolone due to significant and ongoing raised transaminase levels. Abdominal ultrasounds did not detect any form of liver injury and serum alpha fetoprotein concentrations were within the normal range in all children post–onasemnogene abeparvovec administration.

Transient decreases in platelet counts were observed in all infants at approximately 7 days post onasemnogene abeparvovec (Fig. 1A) with no active bleeding. Thrombocytopaenia (platelet count <75 × 10^9^/L) was observed in 7/21 (33%), with the lowest values ranging from 11 × 10^9^/L to 63 × 10^9^/L and recovery occurring by 4–6 weeks post dosing. 6/7 (85.7%) of this subgroup were ≥8 kg at the time of dosing. For the five infants who continued nusinersen post–onasemnogene abeparvovec, platelet values remained in the normal range at 6 months post‐dosing (median 222 × 10^9^/L, range 187 × 10^9^/L–326 × 10^9^/L) and there were no additional safety signals.

At baseline, for those with available troponin I concentrations, 7/11 (64%) had readings above the laboratory reference (median 36 ng/L, range 4–82 ng/L) and were not clinically significant. One‐week post‐dosing, the median change from baseline was 23 ng/L, range 1–49 ng/L). Increased concentration of troponin I was not associated with any clinical abnormalities or ECG changes.

Thrombotic microangiopathy (TMA) occurred in two infants formerly treated with nusinersen. Both infants presented 1 week after onasemnogene abeparvovec infusion with vomiting, transaminitis, thrombocytopaenia, haemolytic anaemia, and acute kidney injury.^14^ One infant had an interval of 4 weeks between sequential therapies and had scheduled immunisation 10 days prior to dosing with onasemnogene abeparvovec. The second child neither had vaccinations or viral illness within 3 months of dosing. A supportive care pathway was followed, including strict fluid management, hypertension control and administration of intravenous steroids. Due to our first TMA case and potential associations with pre‐existing defects in complement pathways, complement pathway testing (C3, C4, CH50, complement factor H and I, CD46, anti‐CFH antibodies and ADAMTS 13) was undertaken before treatment in all subsequent infants. Both infants had reduced C4 and normal C3 levels at the time of presentation of TMA. Complete recovery occurred over 4 weeks following management of symptoms related to the TMA for both patients.

### Clinical efficacy of onasemnogene abeparvovec

Infants were followed for a median of 15 (range 2–26) months post dosing and median age at data cut off was 26 (range 3–42) months. Of the seven infants (33%) requiring NIV at baseline, five (71.4%) continued to require respiratory support following onasemnogene abeparvovec. One infant initiated NIV (and had a fundoplication) following recurrent aspiration pneumonia. All infants with normal oral and swallowing abilities at baseline maintained these skills over the first‐year post‐therapy. For those with bulbar dysfunction at baseline, there was no change in swallowing abilities in 4/7 (67%) over the first year following onasemnogene abeparvovec, while improvement occurred in 3/7 (33%). Specifically, two infants with previously severe swallowing impairment progressed to a combination of oral and enteral feeding with one infant achieving 100% and the second infant achieving 50% of their nutrition intake orally.

### Functional and motor outcomes

**Figure 2:**
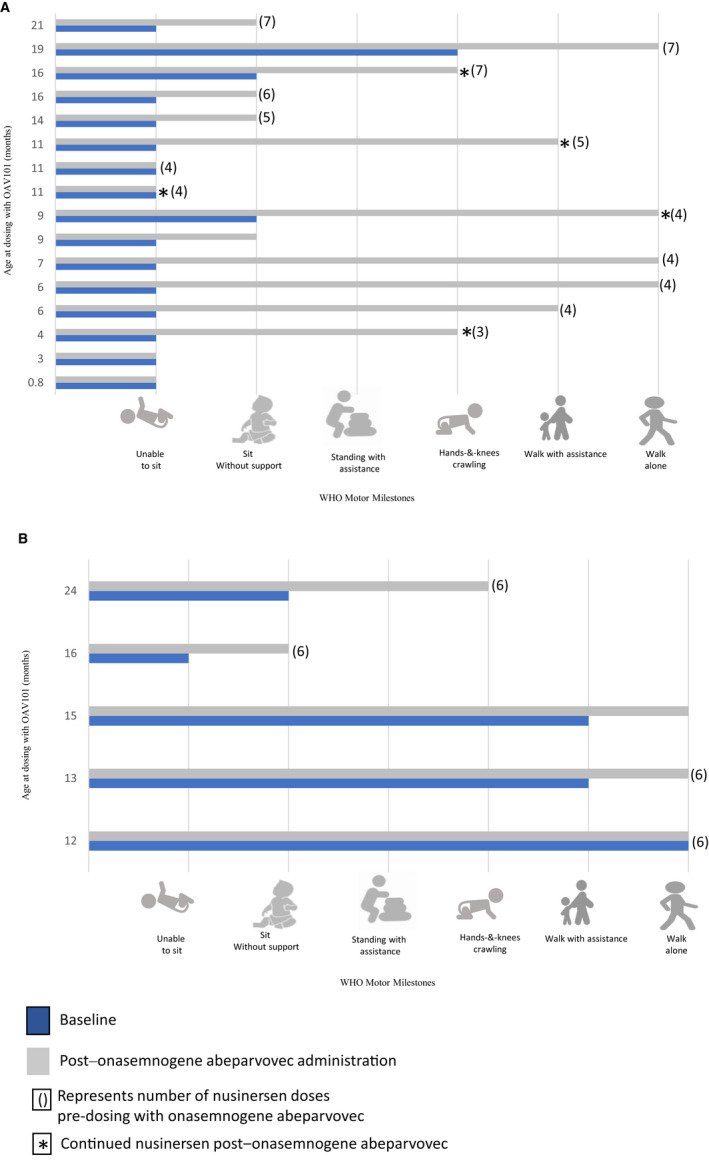
Developmental WHO motor milestones at baseline and following administration of OAV101 in treated children with **(**A) 2 copies of *SMN2* and (B) 3 copies of *SMN2*. WHO developmental assessments were completed for 21 participants at baseline and follow up. Infants were followed for a median of 16 (2–26) months post dosing and median age at data cut off was 2 years (range 0.17 to 3.3 years).^13^ [Colour figure can be viewed at wileyonlinelibrary.com]

As of data cut‐off, 16/21 (76%) infants attained at least one new WHO gross motor developmental milestone, with gains associated to baseline function and clinical manifestations of SMA (Fig. 2). Of the eight non‐sitters, who were beyond the expected developmental age for achieving the ability to sit at baseline, 6/8 (75%) attained “sitting without support” at data cut‐off and 1/8 (12.5%) was also able to “walk with assistance.” There were two infants aged more than 11.5 months at baseline who were “sitting without support” and beyond the developmental age for “standing with assistance.” Both infants attained “hands‐and‐knees crawling” at data cut‐off. One infant was “standing with assistance” aged 19 months at baseline and at data cut‐off attained “walking alone.” WHO gross motor developmental milestones were within the windows for healthy children for six infants with SMA at baseline and at data cut‐off.

As determined by Items 26, 37, and 43 on the BSID3, 11/13 of children (85%) acquired at least one functional motor skill over the study period, with one child treated in the presymptomatic phase gaining additional motor skills that were outside the scope of the developmental assessments utilised in this study.

**Figure 3:**
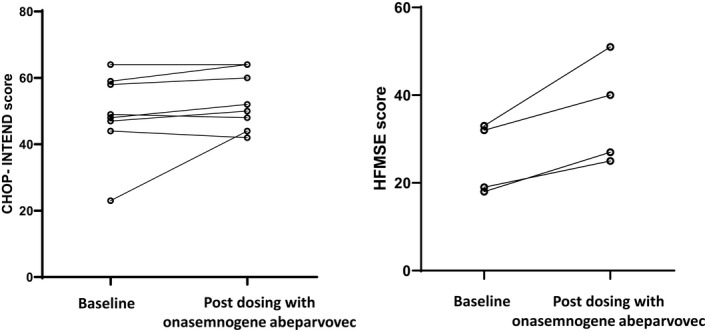
Children’s Hospital of Philadelphia Infant Test of Neuromuscular Disorders (CHOP‐INTEND) values on the left and Hammersmith Functional Motor Scale/ extended version (HFMSE) values on the right in treated children at baseline and 6 months post dosing with onasemnogene abeparvovec.

For children completing validated SMA functional motor assessments (*n* = 13), 9/13 (69%) demonstrated an increase in score over 6 months. An average increase of 10 points (range 6–18) in HFMSE and 7‐point increase (range: 2–21 points) in CHOP INTEND was noted (Fig. 3). One child with substantial muscle weakness and respiratory illness prior to treatment with a low baseline CHOP INTEND score of 49 had an apparent deterioration in motor score by one point.

### Challenges, learnings and parental decision making for onasemnogene abeparvovec

There were substantial challenges for the implementation and integration of the GMAP into the existing neuromuscular service framework (Table 2). Exchange of expertise between stakeholders and establishing standard protocols and streamlining patient pathways for access were imperative to overcome barriers of expansive geography, support logistics for a centralised treatment framework and maintain high‐quality and safe patient‐centred care during the uncertainties associated with the COVID‐19 pandemic.

**Table 2:** Challenges and implementation actions to implementation of a model of care for onasemnogene abeparvovec treatment for SMA in health practice.

Challenges	Implementation action
Differences in modality of diagnosis and baseline function	Support actions for national newborn bloodspot screening programme
AAV9 Ab testing Collection and processing of sample Transport of sample to international lab during COVID 19	Establish standard operating procedures for AAV9 Ab testing (request forms, tubes, collection and processing) Facilitation of local collection to avoid significant travel to a central pathology service Coordination of courier services and customs clearances
Communication and co‐ordination with multiple stakeholders across multiple global time zones for supply and shipping	Maintaining communication (electronic and teleconference) Completion of complex (bi or tripartite) legal agreements SCHN chief executive and drug committee support and approvals
Complex geographical location of patients	Collaboration with interstate neurologists and rural healthcare services Telehealth Education of HCPs and families Emergency letters Stress steroid plan
COVID 19 travel restrictions, quarantines, and NSW health policies enforcements	Psychosocial support for anxiety Coordination of interagency travel permits and quarantine exemptions Allowances for 2 caregivers during medical appointments.
Workforce resources to support national treatment centre	Creation and appointment of gene therapy clinical nurse consultant position to support establishing the service Coordination of treatment pathways, quality improvement activities and translation of research into clinical practice Pharmacist support Dedicated pharmacy facilities
Waste management of GMO’s	Collaboration with on‐site waste disposal departments in‐line with organisational Policy and Procedure requirements Facilitation of appropriate delivery and collection of GMO waste receptacle

SCHN, Sydney Children’s Hospital Network; HCP, Health Care Practioners; GMO, Genetically Modified Organism.

## Discussion

This study characterises the real‐world efficacy and safety of onasemnogene abeparvovec for infants aged 0.65–24 months, weighing 2.5–12.5 kg and thus extending beyond the scope of current real‐world and clinical trial data. The latter is associated with strict inclusion criteria including genotype (mainly focussing on two copies of the *SMN2* gene), weight <8 kg and no prior disease‐modifying treatment. In contrast, our cohort is unique in that it includes older children, who were administered a higher vector load as dependent on their weights. Importantly, our findings relate to children receiving sequential or combination disease‐ modifying agents in the context of a spectrum of disease severity. In addition, we have investigated the parental rationale for accessing therapeutic interventions and ascertained where realistic therapeutic expectations should be placed. This study also describes the barriers and facilitators for administration of onasemnogene abeparvovec, within a dynamic therapeutic landscape, amid a global pandemic.

The therapeutic repertoire for SMA has rapidly advanced and increasingly questions abound regarding the efficacy of individual, sequential or combinatorial treatment options. As there are no head‐to‐head comparative trials, direct comparisons of efficacy between nusinersen and onasemnogene abeparvovec are not possible. Our study showed that the majority of children continued to gain meaningful motor skills over the first year following nusinersen and onasemnogene abeparvovec in various regimens. Although, motor gains were observed for those with lower baseline function, these children continued to have substantial motor impairment. This study emphasises the need to understand the unique constellation of a child's phenotype and baseline functional parameters to help set therapeutic expectations for management. Our study did not include children with long disease duration and markedly low motor function at baseline (for example, CHOP INTEND <20), which has been reported in other studies that have described outcomes in small number of these patients.^15^, ^16^ Thus, the efficacy of onasemnogene abeparvovec in children with advanced disease requires further evaluation.

Bulbar and respiratory complications are the main cause of morbidity and mortality in infants affected with severe SMA, accordingly clinician and parent rationale for accessing onasemnogene abeparvovec and/or continuing combination therapies in our study was to maximise benefits for the weakest and most severely affected children. Whilst nusinersen has efficacy in improving survival, motor function and prolonging time to ventilation, the Australian experience of children with a severe phenotype has been continued gastrostomy feeding and NIV for the first 2 years of treatment.^17^ Systematic assessments of swallowing, feeding and respiratory support are important in evaluating how disease‐modifying therapies change these outcomes, with clinical improvement observed in some infants receiving combination therapy in the present study.

Currently, there is a lack of clinical trial and real‐world data to determine the efficacy and tolerability of sequential or combined therapies in SMA.^18^, ^19^ Our study starts to address this gap by demonstrating that onasemnogene abeparvovec, may have utility in maintaining bulbar and respiratory function for those with the highest disease burdens (i.e., those who require bulbar and respiratory support at baseline and those with the lowest scores on SMA‐validated functional motor scales). For those requiring supplemental feeding at baseline, a portion gained the ability to commence some form of oral nutrition (Table 3). In addition, for the majority of children with high ventilatory needs at baseline, the degree and duration of ventilatory support reduced over the first 12 months following onasemnogene abeparvovec. These findings suggest the possible additional benefits of onasemnogene abeparvovec, with the potential for a multisystemic impact, over and above benefits derived from CNS‐targeted therapies. Whilst previous studies have noted transient transaminitis in children continuing nusinersen post–onasemnogene abeparvovec, our cohort did not display additional safety signals. The longer‐term effects of combined therapy however require evaluation.^20^

**Table 3:** Baseline clinical and functional characteristics of children treated with onasemnogene abeparvovec.

Patient	Sex	*SMN2* copy Number	Age at diagnosis (days)	Nusinersen doses before onasemnogene abeparvovec	Age at onasemnogene abeparvovec dosing (months)	Weight at onasemnogene abeparvovec dosing (kgs)	Baseline respiratory support	Baseline feeding support
1	M	2	9	3	4	7.7	Nil	Oral
2	F	2	14	4	6	7.7	Nil	Oral
3	M	2	19	4	6	6.7	Nil	Oral
4	F	2	17	4	7	7	Nil	Oral
5	F	2	112	4	9	8.2	Nil	Oral
6	M	2	150	4	11	8	NIV	NJT
7	F	2	201	4	11	8.1	Nil (suction)	NGT
8	F	3	15	6	12	12.5	Nil	Oral
9	F	3	13	6	13	9.5	Nil	Oral
10	F	2	166	6	16	9.2	NIV	NGT/oral
11	F	3	130	6	16	9.8	weaning NIV	Oral
12	M	2	20	7	19	9.8	Nil	Oral
13	F	2	106	7	21	9.8	NIV	Oral
14	F	3	329	6	24	11.5	Nil	Oral
15	M	2	27	5	11	9.2	NIV	NGT
16	F	3	10	0	15	11.8	Nil	Oral
17	F	2	48	7	16	8.7	Nil	Oral/PEG
18	M	2	42	5	14	9.1	Nil	NGT
19	F	2	56	4	9	8.8	NIV	NGT
20	M	2	21	3	3	6.2	NIV	Oral
21	F	2	13	0	0.65	2.5	Nil	Oral

M, male; F, female; NIV, non‐invasive ventilation; NJT, nasojejunal tube; NGT, nasogastric tube; PEG, percutaneous endoscopic gastrostomy.

Whilst transaminitis and thrombocytopaenia were observed across our cohort as seen in prior studies,^6^, ^21^ we observed a greater extent of liver and platelet dysfunction in children weighing ≥8 kg. We also observed a bimodal rise in liver enzymes with one peak occurring relatively acutely following therapy. We postulate that the mechanism underlying this early transaminase elevation differs from the later peak which has been attributed to host adaptive immune responses.^20^, ^22^ Given the high vector doses involved, transient direct vector‐mediated hepatotoxicity is plausible and merits further investigation. Close clinical and laboratory monitoring was especially important 2–4 weeks post‐dosing, which coincided with the greatest magnitude of change from baseline in these parameters. Careful safety monitoring was tailored to individuals, with longer close follow‐up of transaminitis particularly for patients weighing ≥8 kg. All children remained asymptomatic despite the changes in blood results, with effective co‐administration of individualised oral prednisolone regime. There was no clinically significant rebound transaminitis or thrombocytopaenia in children post cessation of steroids.

Our study included two children with TMA who completed sequential therapies. Whilst the aetiology of gene therapy‐associated TMA is not fully elucidated; risk factors may include an underlying predisposition to complement activation and/or infections. The contribution of the 10 day interval between routine vaccination and gene therapy in the first infant is uncertain and no putative risk factors were identified in the second case. In preclinical models, ASOs may activate complement pathways.^23^ The combination of nusinersen with onasemnogene abeparvovec may drive inflammation and lead to TMA, with vomiting potentially further contributing to pre‐renal dysfunction. Safety in this context warrants further investigation and will become increasingly relevant as the landscape moves towards combination therapies. Our treatment paradigm changed during the study with a minimum interval of 4 weeks between illness, immunisation, previous disease‐ modifying therapies and onasemnogene abeparvovec dosing. We monitored children closely especially within the first 3 weeks post‐dosing to ensure adequate hydration and tolerance of steroids to reduce the risk of this adverse event.

Preclinical studies involving high doses of AAV vectors have shown dorsal root ganglia damage post–onasemnogene abeparvovec administration.^20^ Whilst electrophysiological testing for sensory neuropathy was not conducted as part of routine clinical practice in this young age group, no child presented with neurologic signs or symptoms of a sensory neuropathy.

Our findings have important ramifications for maintaining safety in children, particularly for older cohorts receiving higher vector loads in line with weight, and for those accessing sequential and combination therapies. Standard guidelines for considering weaning and cessation of steroids is 4 weeks post–onasemnogene abeparvovec administration.^24^ However, based on our findings, we suggest extending dosing, individualising the time and dose of therapy thereafter, dependent on laboratory parameters. Two transaminitis peaks within the first month may be expected and parents should be counselled to this effect. None of our cohort had serious acute liver injury, in contrast to previous studies where hepatic failure and/or severe liver dysfunction necessitated invasive investigation such as liver biopsies.^19^ Whilst asymptomatic thrombocytopaenia is noted in the first week of dosing, in rare cases this can be the first safety signal of TMA. However, the careful observation and monitoring of fluid status, urine output and the evolution of other indices can differentiate TMA from other more benign effects. Although one‐time administration of onasemnogene abeparvovec appears as a “one‐and‐done” procedure, our findings indicate that embedding therapeutic access into a multidisciplinary patient‐centric pathway, to manage long‐term surveillance of safety and efficacy is essential to ascribe true benefits and risks of the intervention and optimise outcomes with standard of care for SMA.^25^ Extended follow‐up will determine whether these benefits are maintained long‐term.

The challenges of implementing high quality and safe treatment to children across a wide sociodemographic and geographical spectrum were multiplied by conducting the program amidst a global pandemic. Coordination of multiple stakeholders including clinical, research, laboratory personnel and consumers was facilitated by establishing operational protocols and sharing expertise across the GMAP network. Embedding these pathways into existing clinical services required consideration of logistical capability, specialised facility requirements for safe storage, handling and disposal of genetic vectors and allocation of a trained workforce. Telehealth played a vital role in facilitating the equitability of access and standardisation of management for children across state borders, especially considering restrictions imposed by the pandemic.

As we shift to a precision medicine paradigm, our study provides the first steps towards identifying key factors for onasemnogene abeparvovec treatment choice (as monotherapy or with sequential therapies). We recognise children at potential risk of adverse reactions to gene therapy including those who weigh ≥8 kg at baseline and potentially for those progressing to sequential therapy. Parental hopes for additional clinical benefit, social media platforms sharing experiences and access conditions have elicited ad hoc combination therapy regimens. Evidence of safety and efficacy will be important to inform the evolving treatment algorithm and reimbursement. As shown in our study, safety and tolerability of onasemnogene abeparvovec was greatest in children weighing <8 kg. This supports the rationale for newborn screening for SMA and early access to onasemnogene abeparvovec, which, to date, has been solely predicated on clinical gains seen in presymptomatic infants.

The study of immune responses in AAV trials has resulted in important advances. However, the complex interactions of AAV with the host immune system need further elucidation, to mitigate safety risks, especially for the severest sequelae. Incorporating these aspects into future clinical trial designs and vector development will boost our knowledge of vector immunogenicity in SMA. This will be key to devise strategies aimed at reliably achieving safe and long‐lasting therapeutic efficacy following AAV vector delivery.

As onasemnogene abeparvovec becomes more accessible to wider populations outside the robust guidelines affiliated with clinical trials and approved regulatory pathways, our study provides an evidence base to balance risks and uncertainties of onasemnogene abeparvovec, support informed therapeutic choice for clinicians and families of children with SMA, and develop health system readiness as the landscape shifts towards providing these advanced therapeutics.

## Author Contributions

AD and MF planned the manuscript. AD, SH, KH and MF collected the data. AD, SH, DK and MF contributed to analysis. AD executed and prepared the first and subsequent drafts of the manuscript. AD, SH, DK and MF contributed to manuscript revision. All authors read and approved the submitted version.

## Conflicts of Interest

Dr. Farrar and Prof. Ryan have received compensation as a member of scientific advisory boards for Biogen, Roche, and Novartis Gene Therapies. Sandra Holland has received compensation as a member of the scientific advisory board for Novartis Gene Therapies.

All other authors declare no potential conflict of interest. These funding bodies had no role in the design of the study, data collection, data analysis, manuscript design, preparation of the manuscript or decision to publish. The authors declare that the research was conducted in the absence of any commercial or financial relationships that could be construed as a potential conflict of interest.
